# Suberoyl bis-hydroxamic acid enhances cytotoxicity induced by proteasome inhibitors in breast cancer cells

**DOI:** 10.1186/s12935-014-0107-7

**Published:** 2014-11-12

**Authors:** Xinmiao Yang, Zeliang Shi, Ning Zhang, Zhouluo Ou, Shen Fu, Xichun Hu, Zhenzhou Shen

**Affiliations:** Department of Radiation Oncology, Shanghai Jiao Tong University affiliated Sixth People’s Hospital, 600 Yi Shan Road, Xuhui District Shanghai, 200233 China; Department of Medical Oncology, Minhang Branch of Fudan, University Shanghai Cancer Center, Shanghai, China; Department of Breast Surgery, Breast Cancer Institute, Shanghai Cancer Center, Fudan University, Shanghai, China; Department of Medical Oncology, Shanghai Cancer Center, Fudan University, Shanghai, China

**Keywords:** Anticancer therapy, Bcl-2 family, Histone deacetylase, Proteasome inhibition, Synergism

## Abstract

**Background:**

Suberoyl bis-hydroxamic acid (SBHA) is a histone deacetylase (HDAC) inhibitor and exerts anti-growth effects in several malignancies including breast cancer. Proteasome inhibitors such as Bortezomib and MG-132 constitute novel anticancer agents. In this study, we investigated the synergistic antitumour activity of SBHA in combination with proteasome inhibitors.

**Methods:**

MCF-7 and MDA-MB-231 breast cancer cells were treated with SBHA, Bortezomib, and MG-132 alone or in combination for 72 h. Cell proliferation, colony formation, apoptosis and gene expression changes were examined.

**Results:**

SBHA, Bortezomib, and MG-132 alone significantly inhibited the proliferation and colony formation and induced apoptosis in MCF-7 and MDA-MB-231 cells. Combined treatment showed a good synergistic antitumour effect against breast cancer cells. The p53 protein level was significantly elevated by combined treatment with SBHA and proteasome inhibitors. Moreover, combined treatment increased the expression of Bax, Bcl-xS, and Bak and decreased the expression of Bcl-2. Combination of SBHA with proteasome inhibitors causes synergistic anticancer effects on breast cancer cells. The potential molecular mechanism may involve induction of p53 and modulation of the Bcl-2 family proteins.

**Conclusion:**

These findings warrant further investigation of the therapeutic benefits of combination of SBHA with proteasome inhibitors in breast cancer.

**Electronic supplementary material:**

The online version of this article (doi:10.1186/s12935-014-0107-7) contains supplementary material, which is available to authorized users.

## Introduction

Breast cancer is one of the most common malignant diseases affecting females worldwide, with more than 450,000 deaths each year [1]. The current treatment modalities for breast cancer include surgical resection, adjuvant radiotherapy, and advanced chemotherapeutic agents such as cisplatin, pacliataxel, carboplatin, bevacizumab, doxorubicin, cyclophosphamide, docetaxel, and epirubicin [2]. Despite advances in treatment strategies, mortality from breast cancer is still high. Combination therapy is gaining increasing attention due to increased antitumor efficacy [3,4].

Histone acetyltransferases (HATs) and histone deacetylases (HDACs) are known to play an opposite role in the regulation of global gene expression via an epigenetic mechanism [5]. HATs catalyze the acetylation of lysine residues in histone tails, facilitating and sustaining gene transcription, while HDACs are responsible for the removal of acetyl groups from the epsilon-amine of lysine residues of histone tails, culminating in prevention of gene transcription. HDAC inhibitors that have the ability to block the activities of HDACs have emerged as effective anticancer agents [6,7]. Suberoyl bis-hydroxamic acid (SBHA) has a similar structure to suberoylanilide hydroxamic acid (SAHA) and trichostatin A (TSA), two of the mostly studied HDAC inhibitors. SBHA has been found to exert anti-growth effects in several malignancies including breast cancer [8].

Proteasome inhibitors such as Bortezomib and MG-132 constitute novel anticancer agents [9]. It has been suggested that proteasome inhibitors interfere with the ubiquitin-proteasome pathway that is involved in protein turnover, likely leading to the accumulation of negative regulators of cell growth and survival in cancer cells. Bortezomib has been found to affect the expression of a large number of genes, especially key regulators of apoptosis such as tumor suppressor protein p53 and Bcl-2 family proteins [10]. Preclinical studies have demonstrated the antitumor activity of Bortezomib in breast cancer [11]. MG-132 exposure also induces cytotoxic effects on a variety of cancer cells including breast cancer cells [12]. Combined treatment with HDAC inhibitors and proteasome inhibitors has been reported to enhance anticancer effects compared to each reagent alone [13,14].

In this study, we aimed to check whether the combination of SBHA with proteasome inhibitors could cause synergistic inhibitory effects on breast cancer cell growth and survival. The molecular pathways involved were also explored.

## Materials and methods

### Cells and reagents

Two human breast cancer cell lines (MCF-7 and MDA-MB-231) and one immortalized breast epithelial cell line (MCF10A) were purchased from American Type Culture Collection (ATCC, Manassas, VA, USA). Fetal bovine serum (FBS), RPMI 1640 medium, DMEM/F-12 medium, and TRIzol reagent were purchased from Invitrogen (Carlsbad, CA, USA), dimethyl sulfoxide (DMSO) from Sigma (St. Louis, MO, USA), SBHA, Bortezomib and MG-132 from Calbiochem (San Diego, CA, USA), Cell Counting Kit-8 (CCK-8) from Dojindo Molecular Technologies (Dojindo, Japan), First Strand cDNA synthesis kit from Fermentas (Burlington, Canada), Annexin-FITC kit from Beckman Coulter (Fullerton, CA, USA), and Apoptotic DNA Ladder Kit (Beyotime, Nantong, China). Antibodies anti-p53, anti-Bcl-2, anti-Bax, and anti-β-actin were purchased from Santa Cruz Biotechnology (Santa Cruz, CA, USA) and anti-Bak and anti-Bcl-Xs from Calbiochem. Horseradish peroxidase-conjugated goat anti-mouse IgG antibody was obtained from Rockland (Gilbertsville, PA, USA).

### Cell culture and treatment

MCF-7 and MDA-MB-231 cells were cultured in RPMI 1640 medium supplemented with 10% heat-inactivated FBS, penicillin (100 units/ml), and streptomycin (100 μg/ml). MCF10A cells were cultured in DMEM/F-12 medium containing 10% FBS, 100 units/ml penicillin and 100 μg/ml streptomycin. They were subcultured every 3-4 days. Twenty-four hours after plating, cells were treated with SBHA (40 μM), Bortezomib (5 nM), and MG-132 (250 nM), alone or in combination, for 72 h and then examined for cell proliferation, apoptosis and gene expression changes. DMSO-treated cells were used as control. To determine the combination index (CI) for combination treatment, MCF-7 and MDA-MB-231 cells were exposed to SBHA plus Bortezomib (a fixed ratio of 8000:1) or SBHA plus MG-132 (a fixed ratio of 160:1) for 72 h and cell viability was assessed using the WST-8 assay. A series of concentrations of SBHA were used, i.e., 10, 20, 40, and 80 μM.

### WST-8 assay

The effect of SBHA on cell proliferation assays was determined with the WST-8 cell proliferation assay kit. Briefly, cells were seeded in 96-well plates at a density of 5 × 10^3^ cells/well and incubated for 24 h. After drug treatment, cells were incubated for further 2 h in the presence of WST-8 reagent. The absorbance (OD) was measured at a wavelength of 450 nm. The CI was calculated according to the classic isobologram equation [15]; CI values of 1, <1 or >1 indicate additivity, synergism or antagonism.

### Colony formation assay

MCF-7 and MDA-MB-231 cells were plated onto 6-well plates and exposed to SBHA (40 μM), Bortezomib (5 nM), and MG-132 (250 nM), alone or in combination, for 72 h. The cells were replated onto 6-well plates at a density of 500 cells per well. After incubation for additional 14 days, cells were washed, fixed in 10% methanol for 15 min, and stained with Giemsa. Colonies consisting of >50 cells were scored. Each experiment was repeated three times.

### DNA ladder assay

DNA was extracted from cells after drug treatment with the Apoptotic DNA Ladder Kit according to the manufacture’s instructions. DNA samples were separated by electrophoresis on 2% agarose gel and visualized by ethidium bromide staining.

### Apoptosis analysis by annexin-V/PI staining

After drug treatment, cells were harvested through trypsinization, washed, and centrifuged at 1,000 r/min for 5 min. The cell pellet was resuspended in 1 × binding buffer. The cell sample solution (100 μl) was incubated with 1 μl of fluorescein isothiocyanate (FITC)-conjugated annexin V and 5 μl of PI for 15 min at 4°C in the dark. The 1 × binding buffer (400 μl) was added to each sample tube and the samples were analyzed on a FACSCalibur flow cytometer using CellQuest software (BD Biosciences, San Jose, CA, USA).

### Reverse transcription-polymerase chain reaction (RT-PCR) analysis

Total RNA was extracted from cells with TRIzol reagent according to the manufacturer’s instructions. Complementary DNA (cDNA) was synthesized with the First-Strand cDNA Synthesis Kit for RT-PCR. Amplification of p53 cDNA was achieved with the following primers: forward 5′-CAGTCAGATCCTAGCGTCGAG-3′ and reverse 5′-TGCAAGTCACAGACTTGGCTGT-3′ (product size, 352 bp). For loading control, glyceraldehyde-3-phosphate dehydrogenase (GAPDH) was amplified in a parallel reaction, with the following primers: forward 5′-GGGAGCCAAAAGGGTCATCATCTC-3′ and reverse 5′-CCATGCCAGTGAGCTTCCCGTTC-3′ (product size, 353 bp). RT-PCR products were separated by electrophoresis on 1.2% agarose gels.

### Western blot analysis

After treatment, cells were lysed in lysis buffer (10 mmol/L Tris, pH7.4, 130 mmol/L NaCl, 1% Triton, 10 mmol/L NaF, 10 mmol/L NaPi, 10 mmol/L NaPPi, and 1.5 mmol/L EDTA) supplemented with protease and phosphatase inhibitors. The protein samples were separated on polyacrylamide gels and then transferred to a nitrocellulose membrane. After blocking for 45 min in a Tris buffered solution (TBS) containing 5% fat-free dried milk and 0.5% Tween-20, the membrane was incubated with individual primary antibodies overnight at 4°C. The membrane was washed three times and incubated for 1 h with secondary antibodies at room temperature. The signals were visualized with the enhanced chemiluminescence method. Densitometric analysis of Western blots was performed using the Scion Image Beta 4.02 software (SynGene, Cambridge, UK).

### Statistical analysis

All data were expressed as mean ± standard deviation (SD). Statistical significance was determined using the Student’s *t*-test or one-way analysis of variance with Tukey's post-test. *P* values less than 0.05 were considered statistically significant.

## Results

### Combination of SBHA and proteasome inhibitors inhibits cell viability and colony formation of breast cancer cells

**Figure 1:**
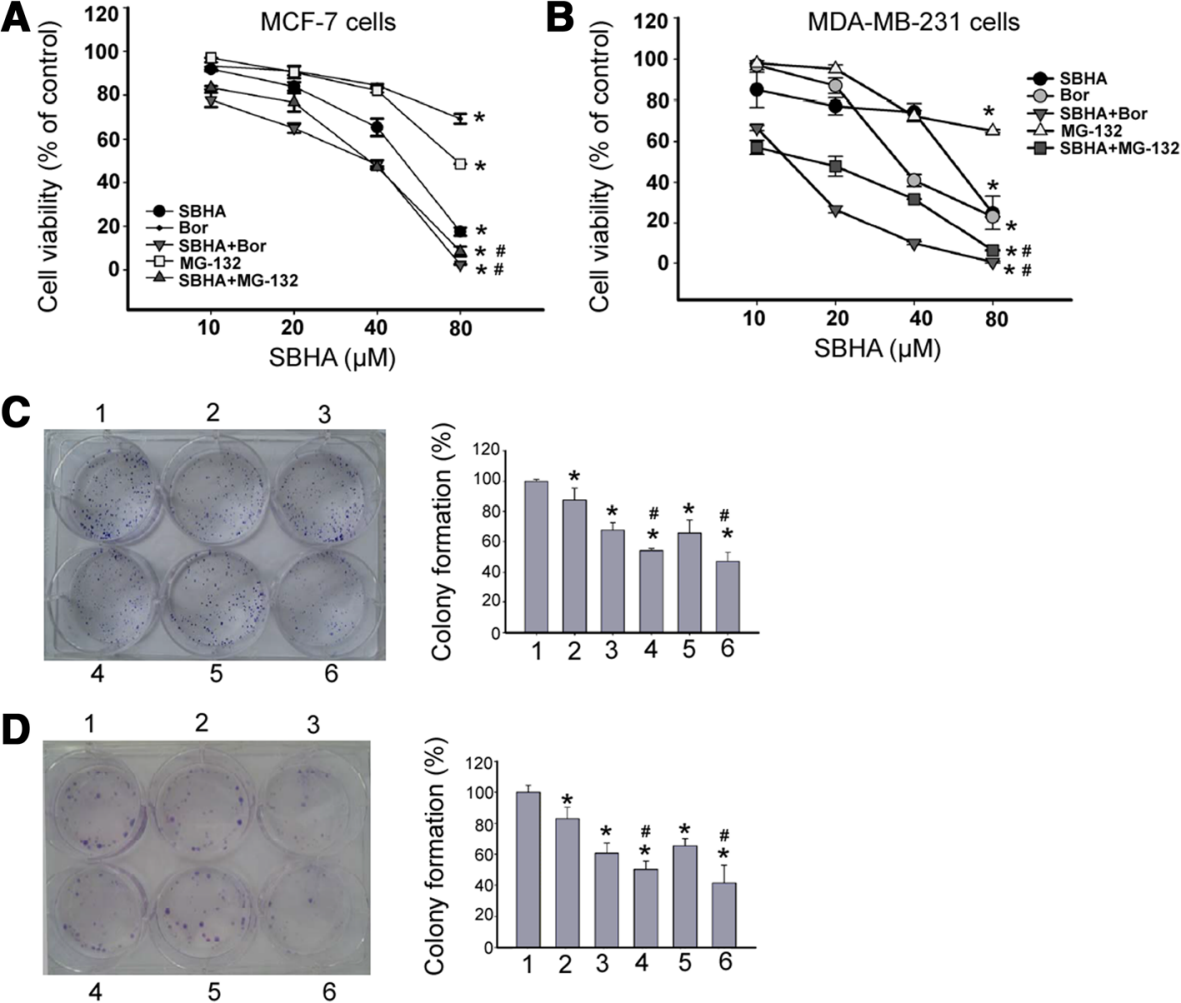
**Effects of combined treatment with SBHA and proteasome inhibitors on breast cancer cell growth. (A)** MCF-7 and **(B)** MDA-MB-231 cells were treated with SBHA, Bortezomib, and MG-132 alone or in combination for 72 h and cell proliferation was assessed using the WST-8 assay. The concentration of Bortezomib and MG-132 was 8000- and 160-fold times that of SBHA, respectively. The proliferation of untreated control cells was considered as 100%. ^*^ *P* <0.05 vs. control; ^#^ *P* <0.05 vs. each reagent alone. **(C)** MCF-7 and **(D)** MDA-MB-231 cells were exposed to SBHA (40 μM), Bortezomib (5 nM), and MG-132 (250 nM), alone or in combination, for 72 h and plated onto 6-well plates at a density of 500 cells per well. Colonies were numbered after 14-day incubation. Left panel: Representative dishes of cells of each group stained with Giemsa. Right panel: Quantitation of colony formation. Colony formation rate was calculated as percentage of total seeded cells. 1-6: control, SBHA, Bortezomib, SBHA + Bortezomib, MG-132, and SBHA + MG-132 group, respectively. ^*^ *P* <0.05 vs. control; ^#^ *P* <0.05 vs. SBHA alone.

WST-8 assay demonstrated that SBHA treatment for 72 h significantly (*P* <0.05) inhibited the proliferation of MCF-7 (Figure 1A) and MDA-MB-231 (Figure 1B) cells, compared to control cells. When SBHA was combined with Bortezomib, greater anti-proliferation effects were achieved (Figure 1A and B). The CI for this combination treatment was 0.60 in MCF-7 cells and 0.57 in MDA-MB-231 cells. The combination of SBHA with MG-132 also exerted a nearly addictive inhibitory effect on breast cancer cell proliferation, with the CI value of 0.97 in MCF-7 cells and 0.42 in MDA-MB-231 cells. To evaluate the synergistic cytotoxicity of SBHA and proteasome inhibitors, the non-malignant MCF10A breast epithelial cells were treated with SBHA (40 μM), Bortezomib (5 nM), and MG-132 (250 nM), alone or in combination. The WST-8 and LDH assays revealed that combined SBHA and Bortezomib or MG-132 had modest adverse effects on MCF10A cell survival (Additional file 1: Figure S1). Therefore, the combination of SBHA with proteasome inhibitors may yield specific inhibitory effects on cancer cells.

To further explore the effects of combination of SBHA and proteasome inhibitors on breast cancer cell growth, colony formation assay was done. Colonies were counted after 14-day incubation. As illustrated in Figure 1C, treatment with SBHA or proteasome inhibitors alone significantly (*P* <0.05) decreased the colony formation of MCF-7 cells, compared to DMSO-treated cells. Notably, combined exposure to SBHA and Bortezomib or MG-132 resulted in significantly greater inhibition of colony formation (Figure 1C). Similar findings were obtained in MDA-MB-231 cells treated with SBHA alone or in combination with Bortezomib or MG-132 (Figure 1D).

### Combination of SBHA and proteasome inhibitors induces apoptosis in breast cancer cells

**Figure 2:**
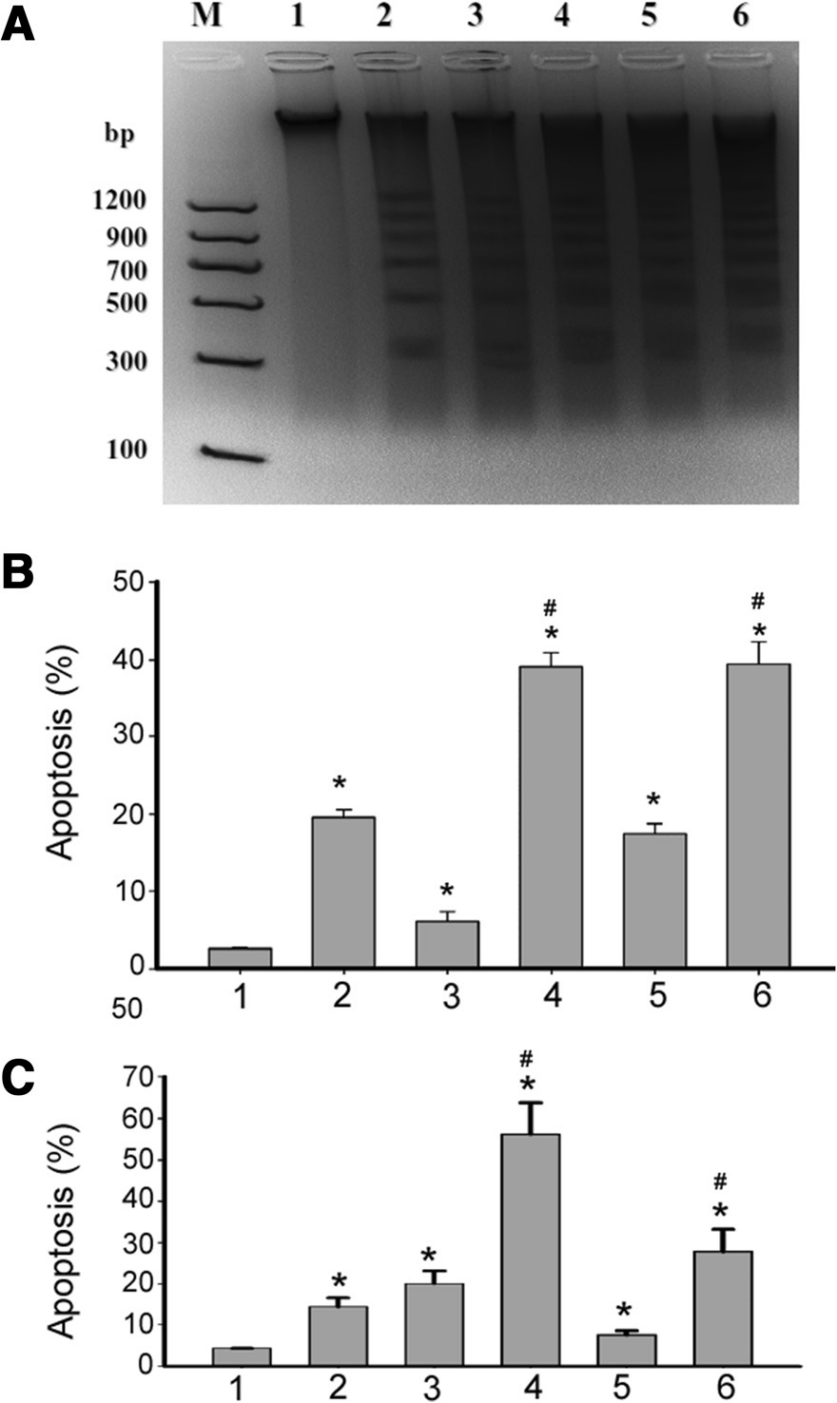
**Effects of combined treatment with SBHA and proteasome inhibitors on breast cancer cell apoptosis.** MCF-7 cells were exposed to SBHA (40 μM), Bortezomib (5 nM), and MG-132 (250 nM), alone or in combination, for 72 h and cell apoptosis was examined. **(A)** Detection of DNA fragments via the DNA ladder assay. Representative image of DNA fragmentation are shown. **(B)** MCF-7 and **(C)** MDA-MB-231 cells were treated with SBHA, Bortezomib, and MG-132 alone or in combination, and cell apoptosis was assessed by annexin V staining assay. Results are expressed as mean ± SD of three independent experiments. Lane 1: molecular-weight marker; lanes 2-6: control, SBHA, Bortezomib, SBHA + Bortezomib, MG-132, and SBHA + MG-132 group, respectively. ^*^ *P* <0.05 vs. control; ^#^ *P* <0.05 vs. each reagent alone.

DNA ladder assay revealed that DNA ladder appeared in MCF-7 cells treated with SBHA, Bortezomib, and MG-132 alone or in combination (Figure 2A). In contrast, DMSO-treated cells did not show typical DNA ladder. For further quantitation of apoptosis, cells were stained with annexin-V and PI and analyzed by flow cytometry. As shown in Figure 2B, treatment with SBHA, Bortezomib, and MG-132 alone caused a significant apoptosis in MCF-7 cells relative to DMSO-treated cells (*P* <0.05). Moreover, the combination of SBHA with Bortezomib- or MG-132 significantly (*P* <0.05) enhanced apoptotic death compared to each agent alone. Similarly, combined treatment with SBHA and Bortezomib- or MG-132 caused a significant (*P* <0.05) induction of apoptosis of MDA-MB-231 cells, compared to each agent alone (Figure 2C).

### Combined exposure of MCF-7 cells to SBHA and proteasome inhibitors upregulates p53 expression

**Figure 3:**
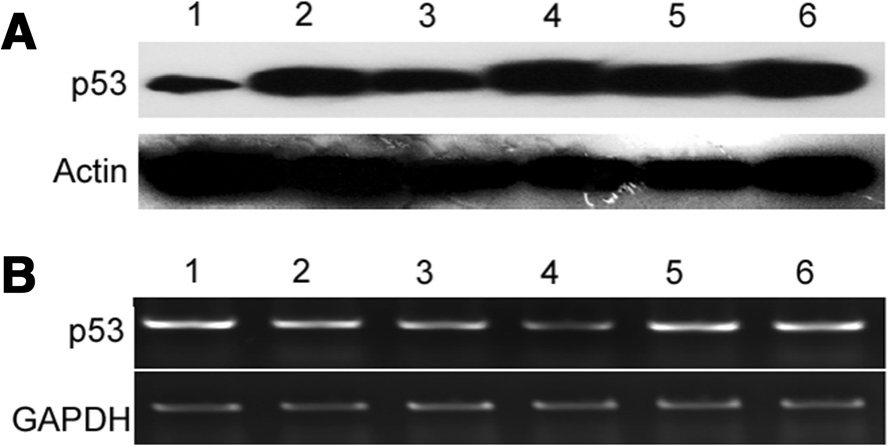
**Effects of combined treatment with SBHA and proteasome inhibitors on p53 expression in breast cancer cells.** MCF-7 cells were exposed to SBHA (40 μM), Bortezomib (5 nM), and MG-132 (250 nM), alone or in combination, for 72 h and p53 expression changes were examined. **(A)** Western blot analysis of p53 protein. Representative blots of three independent experiments with similar results are shown. **(B)** Representative gel images of RT-PCR analysis of p53 transcript are shown. Lanes 2-6: control, SBHA, Bortezomib, SBHA + Bortezomib, MG-132, and SBHA + MG-132 group, respectively.

Western blot analysis revealed that treatment with SBHA, Bortezomib, and MG-132 alone elevated the protein level of p53 in MCF-7 cells relative to DMSO-treated cells (Figure 3A). The p53 protein level was further upregulated when SBHA was combined with Bortezomib or MG-132. However, the p53 mRNA abundance remained unchanged in each treatment group compared to control (Figure 3B).

### Combined treatment with SBHA and proteasome inhibitors affects the Bcl-2 family proteins

**Figure 4:**
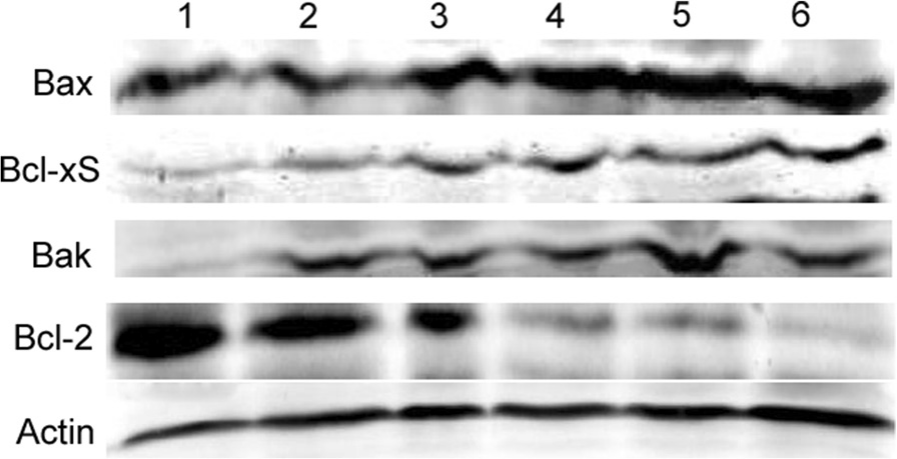
**Effects of combined treatment with SBHA and proteasome inhibitors on the Bcl-2 family members in breast cancer cells.** MCF-7 cells were exposed to SBHA (40 μM), Bortezomib (5 nM), and MG-132 (250 nM), alone or in combination, for 72 h and the Bcl-2 family proteins were examined. Western blot analysis of indicated proteins. Representative blots of three independent experiments with similar results are shown. Lanes 2-6: control, SBHA, Bortezomib, SBHA + Bortezomib, MG-132, and SBHA + MG-132 group, respectively.

Compared to control cells, MCF-7 cells exposed to SBHA, Bortezomib, and MG-132 alone showed an upregulation of Bax, Bcl-xS, and Bak protein and downregulation of Bcl-2 protein (Figure 4). When SBHA was combined with Bortezomib or MG-132, the deregulation of the Bcl-2 family proteins was enhanced (Figure 4).

## Discussion

HDAC inhibitors have been extensively studied for their anticancer activities. SBHA is a relatively new HDAC inhibitor and shows growth-suppressive effects in several types of cancers including medullary thyroid cancer [16] and lung cancer [17]. Zhuang et al. [8] documented that SBHA induces p53-dependent apoptosis of MCF-7 breast cancer cells. Our present data confirm the anticancer effects of SBHA in breast cancer cells. We found that SBHA treatment significantly inhibited the proliferation and colony formation of MCF-7 cells, compared to DMSO-treated control cells. Similarly, treatment with proteasome inhibitors also caused growth suppressive effects against MCF-7 cells. Most interestingly, combined treatment with SBHA and proteasome inhibitors potentiated the suppression of MCF-7 cell proliferation and colony formation. The CI for combination of SBHA with Bortezomib was 0.60, indicating a synergism. Similar synergistic effects of SBHA and Bortezomib were also observed in MDA-MB-231 cells, with the IC value of 0.57. To the best of our knowledge, this is the first report describing the synergistic effects between HADC inhibitors and proteasome inhibitors in breast cancer. The combination of HDAC inhibitors with Bortezomib has also been found to induce synergistic effects against other types of cancers such as primary effusion lymphoma [18,19].

Apoptosis is known as an active suicidal response that plays an important role in tumor biology [20]. It is characterized by cellular shrinkage without loss of plasma membrane integrity, formation of apoptotic bodies, and nuclear condensation and fragmentation. Maintenance of plasma membrane integrity during apoptosis prevents the onset of an inflammatory response that contributes to tumor progression [21]. Therefore, specific induction of apoptosis represents a preferred strategy for destroying tumor cells. Notably, our results demonstrated that SBHA exposure caused a significant apoptosis in MCF-7 cells, which is consistent with the previous report [8]. Bortezomib has been documented to induce apoptosis in breast cancer cells [22,23]. Krętowski et al. [23] reported that Bortezomib treatment evokes a strong effect on apoptosis in breast cancer cells in hypoxic and normoxic conditions. In agreement with these findings, our data confirmed the pro-apoptotic activity of Bortezomib in breast cancer cells. Likewise, MG-132 also showed apoptosis-inducing activity in breast cancer cells. The combination of SBHA with Bortezomib or MG-132 significantly induced apoptosis of MCF-7 and MDA-MB-231 cells compared to each reagent alone. Taken together, these findings suggest that the synergistic anticancer activity of SBHA and proteasome inhibitors in breast cancer cells is, at least partially, mediated through induction of apoptotic death. Although combined treatment with SBHA and Bortezomib or MG-132 caused significant cytotoxicity against breast cancer cells, these combinations did not markedly affect the survival of the non-malignant MCF10A breast epithelial cells. Therefore, the combination of SBHA with Bortezomib or MG-132 may yield specific inhibitory effects on cancer cells.

p53 actively promotes apoptosis and plays a key role in controlling tumor growth [24]. We found that SBHA-treated MCF-7 cells showed a significant elevation in the p53 protein level, but not the p53 mRNA level, suggesting a posttranscriptional regulation. This result is consistent with the previous study that reported an induction of p53 expression in SBHA-treated breast cancer cells [8]. The p53 degradation is largely mediated by the ubiquitin-proteasome pathway [24]. Proteasome inhibition leads to stabilization of p53 [25]. As expected, MCF-7 cells had a significant increase in the p53 protein level after exposure to Bortezomib or MG-132. However, the mRNA abundance of p53 remained unchanged in Bortezomib or MG-132-treated cells. Most interestingly, the p53 protein level was further elevated in MCF-7 cells with combined treatment with SBHA and Bortezomib or MG-132. These findings suggest that induction of p53 may represent an important mechanism for the synergism between SBHA and proteasome inhibitors in breast cancer; however, additional direct evidence is required to confirm the involvement of the p53 pathway.

p53-dependent induction of apoptosis is causally linked to its transcriptional regulation of many target genes. Bax is a downstream target gene of p53 and mediates p53-dependent apoptosis. It has been documented that Bax deficiency impairs p53-induced apoptosis in neurons [26]. The upregulation of Bax is implicated in HDAC inhibitor-induced apoptosis in breast cancer cells [27]. For instance, Wang et al. [27] reported that sirtinol, a class III HDAC inhibitor, induces apoptotic death in MCF-7 cells through upregulation of Bax. SBHA has also been documented to enhance the expression of Bax in MCF-7 cells, which contributes to p53-dependent apoptosis [8]. In agreement with this study, our data showed that SBHA-treated MCF-7 cells had a significant increase in the Bax protein level. Moreover, the Bcl-xS and Bak proteins were elevated in SBHA-treated cells. When SBHA was combined with Bortezomib or MG-132, the upregulation of Bax, Bcl-xS, and Bak was significantly enhanced. In contrast, the Bcl-2 protein level was deceased upon exposure to SBHA, Bortezomib, and MG-132 alone or in combination. Bax is a pro-apoptotic member of the Bcl-2 family. It undergoes mitochondrial intramembranous homo-oligomerization in response to apoptotic stimuli, which promotes release of cytochrome c from mitochondria, consequently activating the mitochondrial apoptotic pathway [28]. Bcl-xS is localized in the mitochondria and induces apoptosis via activation of Bak [29]. The anti-apoptotic protein Bcl-2 is predominantly localized to mitochondria and can interact with Bax to inhibit its activation [30]. Taken together, our data suggest that the pro-apoptotic activity induced by combined treatment with SBHA and proteasome inhibitors is associated with modulation of the Bcl-2 family members.

SBHA and proteasome inhibitors have shown cytotoxicity in a broad range of cancer types, such as colorectal cancer [31], prostate cancer [13], and lung cancer [17]. Combination treatment with HDAC inhibitor TSA and low-dose Bortezomib has been reported to induce synergistic apoptosis in prostate cancer cells [32]. The addition of HDAC inhibitor SAHA to Bortezomib treatment was found to cause synergistic effects against primary effusion lymphoma cells. These studies, combined with our present findings, suggest that apart from breast cancer, the SBHA/proteasome inhibitor combination therapy may induce synergistic cytotoxicity in other types of malignancies.

In conclusion, our data demonstrate that combination of SBHA with proteasome inhibitors enhances anticancer effects on breast cancer cells through promotion of apoptotic death. Induction of p53 and modulation of the Bcl-2 family proteins at least partially account for the synergism between SBHA and proteasome inhibitors. These findings warrant further investigation of the therapeutic potential of combination of SBHA with proteasome inhibitors in breast cancer.

## Additional file

Additional file 1: Figure S1. Effects of combined treatment with SBHA and proteasome inhibitors on the survival of MCF10A cells. Cells were treated with SBHA, Bortezomib, and MG-132 alone or in combination for 72 h and cell viability and death were assessed. **(A)** Cell viability was determined using the WST-8 assay. The viability of control cells was considered as 100%. **(B)** LDH assay was done to assess cell death. Data represent means ± SD of three independent experiments.
