# Supplementary material for: Suberoyl bis-hydroxamic acid enhances cytotoxicity induced by proteasome inhibitors in breast cancer cells
Source: Cancer Cell Int. 2014 Nov 12;14:107. doi: 10.1186/s12935-014-0107-7 (PMC4342900; doi:10.1186/s12935-014-0107-7)

**Materials and methods**

**Lactate dehydrogenase (LDH) assay**

MCF10A cells were seeded onto 96-well plates at the density of 3 × 10^3^ cells/well. After incubation overnight, cells were untreated or exposed to SBHA (40 μM), Bortezomib (5 nM), and MG-132 (250 nM), alone or in combination. For each treatment, six replicates were used. After incubation for 72 h, the cells were tested for LDH release using the CytoTox 96 nonradioactive cytotoxicity assay (Promega, Madison, WI, USA ) according to the manufacturer's instructions.

**Results**

**Fig. S1. Effects of combined treatment with SBHA and proteasome inhibitors on the survival of MCF10A cells.** Cells were treated with SBHA, Bortezomib, and MG-132 alone or in combination for 72 h and cell viability and death were assessed. (A) Cell viability was determined using the WST-8 assay. The viability of control cells was considered as 100%. (B) LDH assay was done to assess cell death. Data represent means ± SD of three independent experiments.


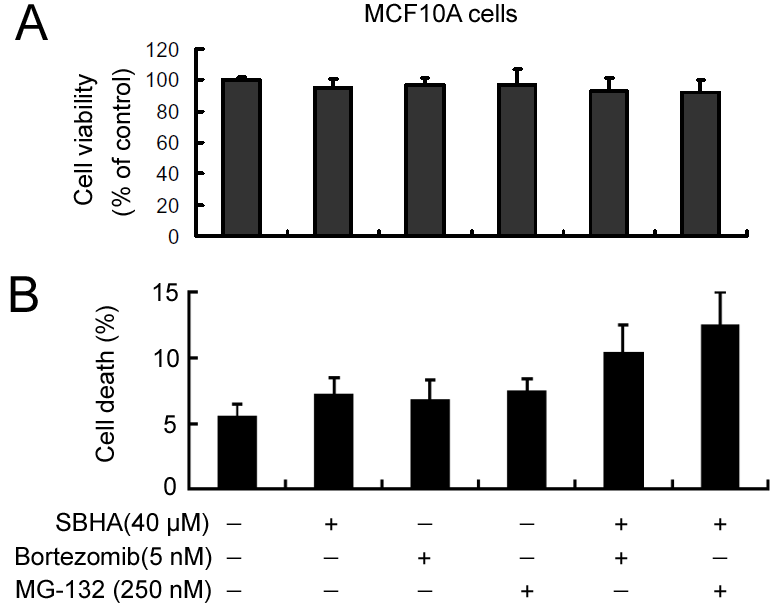

Supplement: Additional file 1: Figure S1. — Effects of combined treatment with SBHA and proteasome inhibitors on the survival of MCF10A cells. Cells were treated with SBHA, Bortezomib, and MG-132 alone or in combination for 72 h and cell viability and death were assessed. (A) Cell viability was determined using the WST-8 assay. The viability of control cells was considered as 100%. (B) LDH assay was done to assess cell death. Data represent means ± SD of three independent experiments. [file 12935_2014_107_MOESM1_ESM.doc]
